# Unraveling the complexity of Disorders of the Gut-Brain Interaction: the gut microbiota connection in children

**DOI:** 10.3389/fped.2023.1283389

**Published:** 2024-02-16

**Authors:** Dimas Rosa, Roberto Arturo Zablah, Rodrigo Vazquez-Frias

**Affiliations:** ^1^ Grupo de Investigación del Caribe y Centroamérica para la Microbiota, Probióticos y Prebióticos, GICCAMPP, la Romana, Dominican Republic; ^2^ Servicio de Gastroenterología y Endoscopia Digestiva, Hospital de Niños “Benjamín Bloom”, San Salvador, El Salvador; ^3^ Departamento de Gastroenterología y Nutrición Pediátrica, Instituto Nacional de Salud Hospital Infantil de México Federico Gómez, Ciudad de México, México

**Keywords:** microbiota, dysbiosis, immunity, inflammatory bowel diseases, probiotics, prebiotics, diversity, microbiome

## Abstract

“Disorders of Gut-Brain Interaction (DGBIs),” formerly referred to as “Functional Gastrointestinal Disorders (FGIDs),” encompass a prevalent array of chronic or recurring gastrointestinal symptoms that notably impact the quality of life for affected children and their families. Recent studies have elucidated the intricate pathophysiology of DGBIs, underscoring their correlation with gut microbiota. This review seeks to explore the present comprehension of the gut microbiota's role in DGBI development. While other factors can contribute to DGBIs, the gut microbiota prominently influences the onset and progression of these conditions. According to the Rome IV diagnostic criteria, DGBI prevalence is approximately 40% worldwide. The Rome Foundation has diligently worked for nearly three decades to refine our comprehension of DGBIs. By centering on the gut microbiota, this review sheds light on potential therapeutic interventions for DGBIs, potentially enhancing the quality of life for pediatric patients and their families.

## 1 Introduction

Disorders of Gut-Brain Interaction (DGBIs), formerly called Functional Gastrointestinal Disorders (FGIDs), are common conditions in clinical practice and in the community. They are defined as “the set of chronic or recurrent gastrointestinal symptoms, not explained by structural or biochemical abnormalities, with significant interference in the quality of life of the child and his family.” (1) The global occurrence of DGBIs stands at approximately 40%, as indicated by a recent survey employing the Rome IV diagnostic criteria (2). Over the course of nearly three decades, the Roma Foundation has aimed to validate and enhance our understanding of DGBIs.

While the intricate pathophysiology of DGBIs is increasingly recognized, with a central role attributed to the gut microbiota, it is essential to highlight the concept of dysbiosis. Dysbiosis, understood as the loss of diversity or the alteration in the balance of the usual composition of the human microbiota, when established from an early age, can predispose to the development of a spectrum of diseases, including gastrointestinal alterations, immune and metabolic diseases, allergies, and even cancer (3) This review, framed as a narrative exploration, seeks to shed light on the current comprehension of the gut microbiota's role in DGBI development. The sources used in this narrative review include peer-reviewed research articles, clinical studies, and relevant publications from reputable scientific journals, with PubMed serving as our primary search platform. The search term employed was “Pediatric Gut-Brain Interaction Microbiota,” ensuring a focused exploration of the literature pertinent to our review.

While we have chosen to focus on the potential role of probiotics as treatment measures, our aim is to provide a specific exploration within the broader landscape of therapeutic interventions for Disorders of Gut-Brain Interaction (DGBIs). Probiotics have garnered attention for their potential impact on the intestinal microbiota and, consequently, on symptoms associated with DGBIs. Recognizing the existence of various therapeutic approaches, including other microbiota modifiers such as prebiotics, symbiotics, or postbiotics, in addition to dietary and lifestyle interventions, our narrative review centers on probiotics to offer a detailed analysis of their potential benefits in the context of pediatric DGBIs. This specific approach allows for an in-depth analysis and discussion of probiotics as a treatment modality within the scope of our review.

## 2 Background

In 2006, the Rome III Criteria were released, introducing two distinct pediatric categories within Functional Gastrointestinal Disorders (FGIDs): Neonates/young children (category G) and children/adolescents (category H). This differentiation stemmed from the varying clinical circumstances that arise between these two categories, influenced by the growth and development of children (4). During that period, limited evidence was accessible concerning the epidemiology, underlying mechanisms, diagnostic procedures, treatment approaches, and subsequent monitoring. As a result, the criteria for these clinical conditions were primarily founded on practical knowledge rather than empirical evidence. Subsequent to that time, there has been a substantial expansion in our scientific comprehension of these disorders. While the full Rome pediatric criteria are not presented here to maintain focus on the microbiota aspect and to avoid redundancy, interested readers are directed to the comprehensive criteria outlined by the Rome Foundation. The publication by Benninga et al. in 2016 encompasses the Diagnostic Criteria for Functional Gastrointestinal Disorders in Neonates and Toddlers (5) and Hyams outlines those for children and adolescents (6).

## 3 General pathophysiology of Disorders of Gut-Brain Interaction (DGBIs)

### 3.1 Biopsychosocial model

The biopsychosocial model has transformed the medical paradigm regarding the origins and mechanisms of functional abdominal pain disorders, shifting it away from solely attributing these disorders to organic causes in an effort to unravel the underlying factors behind these symptoms (7).

The biopsychosocial model includes:

• Sensitizing factors, among which are:
  ○ The balance of gut microbiota can be affected by antibiotics, proton pump inhibitors, antidepressants, corticosteroids, immunosuppressants, laxatives, chemotherapy, and certain cardiovascular drugs, among others.
  ○ Inflammatory factors, allergies, and infections.
  ○ Motility disorders.
• Genetic predisposition
• Psychosocial sensitizing factors
  ○ Pattern of abdominal pain, family stress, abuse.
  ○ Depression, anxiety, and stress.
  ○ Lifestyle modeling and secondary.
  ○ Early stressors.

Today, it is evident that a series of sensitizing factors can influence the composition of the microbiota. Now, it is crucial to comprehend the intricate relationship between the microbiota and metabolism, which ultimately may give rise to disorders in the gut-brain interaction.

### 3.2 Microbiome and metabolism

Discoveries of key characteristics of the microbiome, carbohydrate and amino acid metabolism are providing new insights into potential therapies or preventive strategies for DGBIs. The microbiota of pediatric patients and healthy adults differ in the relative abundance of intestinal bacterial taxa. In adults there is a greater number of Proteobacteria compared to children who also have the presence of Actinobacteria, which are found in a lower proportion in adults (8).

While there was a previous belief that the microbiota began developing *in utero*, it is now evident that it does not initiate there. However, we do know that factors during pregnancy start to shape how the microbiota develops. The initiation of the gut microbiota's formation occurs at birth and is then shaped by dynamic shifts during the early years of life. These shifts encompass a range of environmental and parental influences, including factors like host genetics, mental well-being, dietary habits, delivery method, feeding practices, antibiotic usage, and immune system activity (9).

The gut microbiota and its metabolites influence the programming of the immune system and central nervous system. The first 1,000 days are a very vulnerable period of insults that can cause lasting effects on the microbiota-gut-brain axis. After birth, a newborn's gut microbiota is transiently dominated by *Enterobacteriaceae* and *Staphylococcus.* Subsequently, *Bifidobacterium* and some lactic acid-producing bacteria predominate in a baby's gut microbiota. The microbiota dominated by Bifidobacteria is maintained until the introduction of solid foods (10). Nutrition in early life plays a fundamental role in perinatal programming and in modulating the microbiota of offspring from birth to life (11). Prior to weaning, the early microbiota experiences an enrichment of bacteria possessing genes that aid in the utilization of lactate. Following the transition to solid foods after weaning, there is a promotion of the growth of bacteria enriched in genes that facilitate the utilization of a wider array of carbohydrates, the synthesis of vitamins, and the breakdown of xenobiotics. After weaning, Bifidobacteria are overtaken by adult-type microorganisms, represented mainly by bacteria of the genera *Bacteroides*, *Prevotella*, *Ruminococcus*, *Clostridium* and *Veillonella*, which colonize the intestines of babies. Approximately by the age of three, a characteristic gut microbiota resembling that of adults takes shape. As individuals grow older, the diversity of the gut microbiota progressively expands until it reaches a point of stability in adulthood (9).

### 3.3 Microbiota and gut motility

Impaired motility and gastrointestinal transit have long been recognized in the pathophysiology of DGBIs such as IBS and Functional Dyspepsia. There exists a reciprocal interaction between gastrointestinal motility and the gut microbiota, underscoring a two-way connection. Gut microorganisms can accelerate the pace at which substances move through the gastrointestinal tract. Conversely, expedited movement within the gastrointestinal tract can influence the composition and arrangement of microbial communities by creating favorable conditions for the growth of specific bacterial groups or by affecting bacterial adhesion. Various agents derived from microbes have been recognized as influential factors in gastrointestinal motility. Short-chain fatty acids (SCFAs) and bile acids are among them. SCFAs emerge through the fermentation of dietary starches or complex carbohydrates by gut bacteria, while the modification of bile acids by gut bacteria influences the volume and forms of bile acids in the colon. The promotive effects of bile acids on gut motility might be channeled through the G-protein-coupled bile acid receptor TGR5 (or GPBAR1), which is present in enteric neurons and enteroendocrine cells, as suggested by animal studies. Notably, the microbial agents influencing gastrointestinal motility could vary based on diet; concentrations of SCFAs might differ according to the intake of dietary carbohydrates and proteins. Additional microbial byproducts or metabolites with potential relevance to the microbial control of gastrointestinal motility encompass bacterial lipopolysaccharides, which can enhance the viability of enteric neurons by activating Toll-Like Receptor 4 (TLR4). Preliminary investigations lend support to the roles of other microbial metabolites like methane, hydrogen sulfide, tryptamine, and hydrogen gas in modulating human gastrointestinal motility due to their suggested effects on gastrointestinal smooth muscle and the enteric nervous system (12).

## 4 Physiopathology of the most frequent DGBIs in pediatrics

### 4.1 Infant colic

Comprehending Infant Colic entails recognizing the progression of the infant's growth, the connection with their caregiver, and the broader family and social context within which they are situated (5).

Infant Colic has been characterized as “a behavioral syndrome in 1- to 4-month-old infants involving long periods of crying and hard to soothe behavior. The crying bouts occur without obvious cause so that their unexplained nature is one of the main reasons for caregivers ‘concerns.” (5)

The exact cause of Infantile Colic remains uncertain and is believed to stem from multiple factors. However, an increasing body of evidence indicates that the gut microbiome plays a role in the onset of the condition. Various other factors could potentially contribute to the emergence of infant colic, including neurodevelopmental factors, gastrointestinal aspects, feeding methods, and psychosocial influences (10).

#### 4.1.1 Microbiota composition and Infant Colic

Of all these factors, dysbiosis emerges as one of the factors that in recent times has become very relevant. In terms of microbial diversity, stability, and colonization patterns, the gut microbial phyla of colicky infants exhibit disparities compared to those of non-colic infants (12).

In 2007, Savino et al. conducted a study revealing distinctions in the gut microbiota of infants with and without colic. Infants with colic showed reduced colonization by *Lactobacillus* spp. and a heightened presence of gas-producing anaerobic bacteria, notably *Escherichia coli* (13).

Another comprehensive investigation focused on the fecal microbiota of colicky infants throughout their initial hundred days of life, comparing them to non-colicky control infants. The findings indicated that while the diversity of microbiota gradually increased in the control group post-birth, the diversity of the colicky group was notably lower, particularly in the early weeks. Additionally, the stability of subsequent samples appeared notably diminished among colicky infants in their initial weeks of life. These findings suggest the presence of distinct microbial patterns in the first weeks of life among infants who later develop colic. The typical microbiota of an infant with colic exhibited a positive correlation with specific groups of proteobacteria, with their relative abundance more than doubling. Conversely, *Bifidobacterium* and *Lactobacilli* experienced significant reductions in infants with colic. These microbiological findings have provided valuable information to understand the underlying reasons for colicky babies (14).

In order to delve deeper into the distinctions between infants with colic and those in the control group, the diversity of microbiota was assessed. Among control infants, there was a gradual increase in microbiota diversity over time. On the other hand, the microbiota diversity in colicky infants displayed a distinct temporal development, remaining considerably low during the initial 100 days of life (14).

Various other studies have shed light on the link between the gut microbiome and Infant Colic. Korpela et al. investigated with the objective of exploring the association between the initial meconium microbiome and subsequent occurrences of Infant Colic. The results of the study revealed that infants who later experienced Infant Colic exhibited a diminished relative abundance of the *Lactobacillus* genus and the *Firmicutes* phylum in their initial stool samples, in contrast to those who maintained good health (15).

With reference to colic, it can be concluded that children who present it have a lower diversity of their intestinal microbiome with the presence of a greater number of Proteobacteria, Bacteroidetes and Verrucomicrobia and fewer Firmicutes and Actinobacteria, as well as a lower number of Lactobacilli and Bifidobacteria.

#### 4.1.2 Low-grade inflammation and Infant Colic

Zeevenhooven et al. have delineated various factors encompassing neurogenic, gastrointestinal, microbial, and psychosocial element that could play a role in the development of Infant Colic's pathophysiology. Disruption in gut microbiota might potentially play a role in the manifestation of colic symptoms by intensifying the fermentation of lactose, carbohydrates, and protein. This could lead to an increased production of gas and subsequent bloating. Heightened permeability in the intestines could potentially facilitate an escalation in low-level systemic and mucosal inflammation, which could be influenced by elevated levels of Gram-negative bacteria, including species such as *Escherichia* spp., *Bacteroidetes* spp., and *Klebsiella* spp. Pathogen-associated lipopolysaccharide, a component present in the outer membranes of Gram-negative bacteria, may stimulate the release of pro-inflammatory cytokines and chemokines, thereby inciting a reaction in the intestinal epithelial cells. Elevated concentrations of proinflammatory cytokines, encompassing IL-8, chemokine ligand 2 (CCL2), and CCL4, might potentially indicate the presence of low-level intestinal inflammation in cases of Infant Colic. The intricate microbiota-gut-brain axis could potentially mediate the influence of gut dysbiosis on central and enteric neuronal function, encompassing aspects like pain and crying detection, particularly in infants experiencing colic (10).

Rhoads et al. conducted a study aiming to investigate whether intestinal inflammation, colonic fermentation, and/or alterations in colonic flora could potentially serve as a pathophysiological mechanism behind colic. The study's findings revealed that colicky infants cried and displayed discomfort for twice the duration of the non-colic group. Faecal calprotectin levels were found to be twofold higher in infants with colic compared to the control group. The presence of *Klebsiella* species was more prevalent in colicky patients than in control patients, while *Enterobacter*/*Pantoea* species were exclusively detected in control patients. These outcomes could not be attributed to variations in feeding methods (breast milk vs. formula), elemental formula intake, or antibiotic exposure. Ultimately, the study concludes that infants with colic, a condition previously believed to lack organic underpinnings, exhibit indications of neutrophil infiltration in the intestines and a less diverse composition of fecal microbiota (16).

Another study showed that children with colic have in addition to increased fecal calprotectin an elevated IL-8 level and a reduction in IL-7 (17).

**Figure 1:**
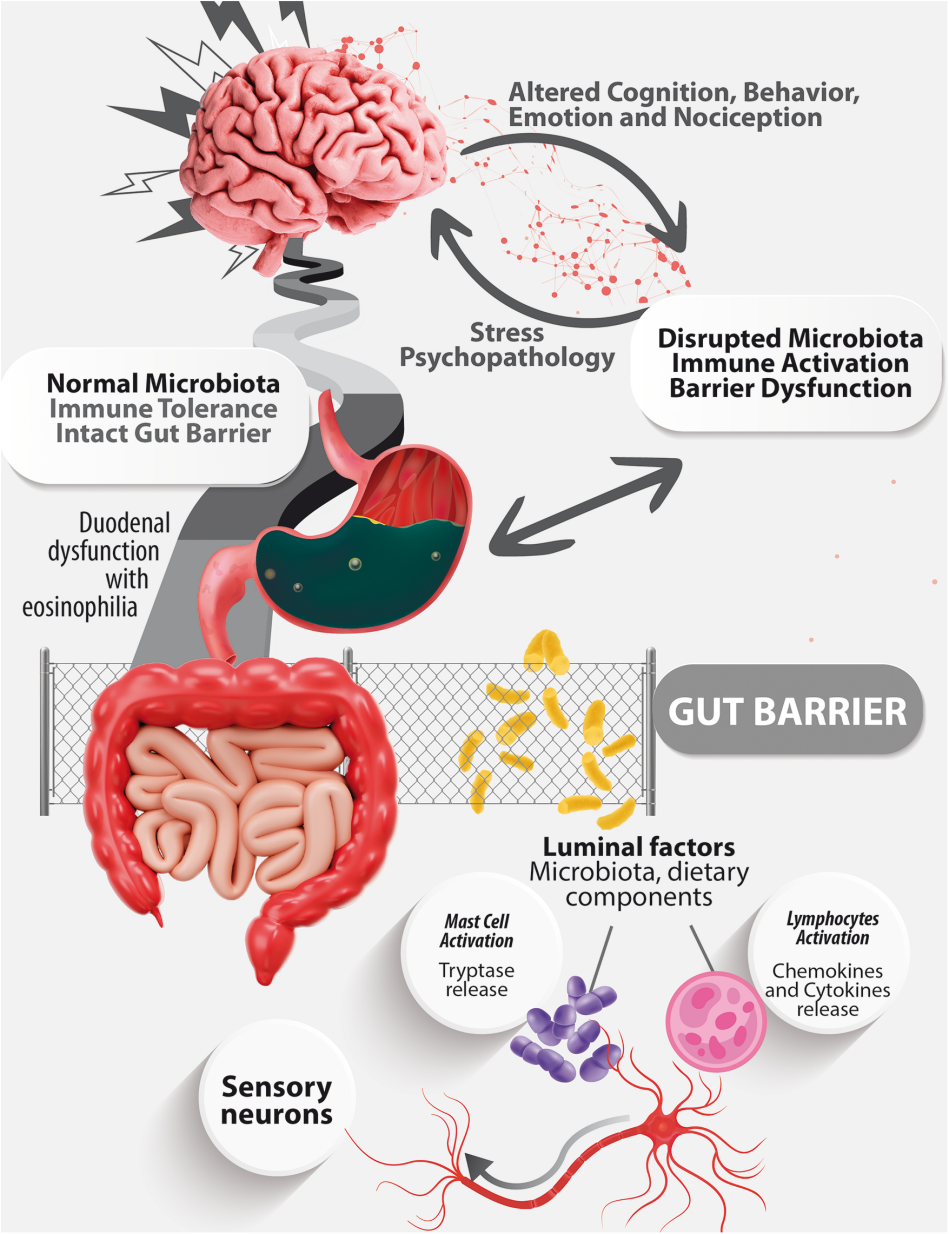
A schematic representation of the microbiota-gut-brain axis.

Figure 1 outlines how Disorders of the Gut-Brain Interaction are marked by compromised brain processing associated to alterations in the gut luminal content, including dysbiosis. It is crucial to emphasize that luminal changes in the small intestine are defined by intestinal inflammation accompanied by visceral hypersensitivity.

#### 4.1.3 Studies of probiotics in Infant Colic

ESPGHAN offers updated guidelines for the utilization of probiotics in addressing specific pediatric gastrointestinal disorders (Table 1). These recommendations were formulated based on a minimum of two Randomized Controlled Trials (RCTs) conducted with a well-defined probiotic strain of similar nature. In the context of Infant Colic, this publication proposes that healthcare professionals may consider suggesting the use of *Limosilactobacillus reuteri* DSM 17938 (at a minimum of 10^8^ CFU/day for a duration of at least 21 days) and *Bifidobacterium lactis* BB-12 in breast-fed infants (at a minimum of 10^8^ CFU/day for 21–28 days) as a treatment for Infant Colic in infants, with a moderate level of evidence and a weak degree of recommendation. Similarly, the publication refrains from offering a decisive recommendation either in favor of or against the use of *L. reuteri* DSM 17938 in formula-fed infants due to a lack of sufficient evidence (18). The World Gastroenterology Organization's (WGO) Probiotics and Prebiotics Guidelines include probiotic strains with a solid level of Oxford evidence in their list (19).

**Table 1:** Evidence-based probiotic recommendations for children with DGBI: leading guidelines (ESPGHAN & WGO).

	ESPGHAN 2023 (18)	WGO 2023 (19)
Infant Colic management	*L. reuteri* DSM 17938 (10^8^ CFU/day for at least 21 days) for the management of infant colic in breastfed infants (certainty of evidence: moderate; grade of recommendation: weak). *B. lactis* BB-12 (10^8^ CFU/day, for 21–28 days) for the management of infant colic in breastfed infants (certainty of evidence: moderate; grade of recommendation: weak). No recommendation can be made for or against the use of *L. reuteri* DSM 17938 in formula-fed infants due to insufficient evidence.	*L. reuteri* DSM 17938 (10^8^ CFU/day for at least 21 days). Oxford evidence level 1. *B. lactis* BB-12 (10^8^ CFU/day, for 21–28 days). Oxford evidence level 2. *L. rhamnosus* 19070-2 and *L. reuteri* 12246 in a daily dose of 250 × 10^6^ CFU 3.33 mg of fructooligosaccharide 250 × 10⁶ CFU respectively, plus 3.33 mg of fructooligosaccharide, for 28 days. Oxford evidence level 3. *L. paracasei* DSM 24733, *L. plantarum* DSM 24730, *L. acidophilus* DSM 24735, *L. delbrueckii* subsp. *bulgaricus* DSM 24734), *B. longum* DSM 24736, *B. breve* DSM 24732, and *B. infantis* DSM 24737, and *S. thermophilus* DSM 24731. Oxford evidence level 3.
Infant Colic prevention	No recommendation can be made for or against the use of any of the probiotics studied so far for preventing infant colic due to insufficient evidence.	*L. reuteri* DSM 17938 10^8^/day, to newborns each day for 90 days. Oxford evidence level 1.
Functional Abdominal Pain Disorders	*L. reuteri* DSM 17938 (at a dose of 10^8^CFU to 2 × 10^8^ CFU/day) for pain intensity reduction in children with functional abdominal pain disorders (certainty of evidence: moderate; grade of recommendation: weak).	Functional abdominal pain/IBS: *L. reuteri* DSM 17938 10^8^/day. Oxford evidence level 1. Oxford evidence level 1. *L. rhamnosus* GG 10^9^ UFC to 3 × 10^9^ CFU twice daily. Oxford evidence level 1.
Functional Constipation	Not recommendation can be made for the use of probiotics as a single or adjuvant therapy for treatment of functional constipation in children due to the lack of efficacy (certainty of evidence: moderate; grade of recommendation: weak).	Not recommendation can be made for the use of probiotics in constipation in children.

Additional strains have also undergone investigation, including a mixture of *Bifidobacterium longum* CECT7894 (KABP042) and *Pediococcus pentosaceus* CECT8330 (KABP041) at a dosage of 1 × 10^9^ colony forming units. The study determined that administering *B. longum* CECT7894 (KABP042) and *P. pentosaceus* CECT8330 (KABP041) orally on a daily basis proved to be an effective intervention for reducing crying duration caused by Infant Colic and for enhancing fecal consistency (20).

In a study involving *Lacticaseibacillus rhamnosus* ATCC 53103, administered concurrently with the exclusion of cow's milk from the maternal diet for a duration of 28 days, intriguing outcomes were demonstrated pertaining to the impact of this probiotic intervention. The study observed reductions in crying duration and fecal calprotectin levels, coupled with an augmentation in total bacterial counts and *Lactobacillus* populations. Nevertheless, in order to substantiate these findings, a double-blind, placebo-controlled trial involving a more extensive participant cohort is necessary (21).

In 2018, a study assessed the impact of a blend of two *Lactobacillus* strains on reducing crying and fussiness in infants exclusively breastfed. The findings indicated that the combination containing *Lactobacillus rhamnosus* 19070-2 and *Lactobacillus reuteri* 12246 led to a reduction in both crying and fussing time, offering dietary support to colicky infants who were exclusively breastfed (22).

### 4.2 Functional Constipation

According to Benninga et al., Functional Constipation frequently arises from repetitive attempts by a child to intentionally withhold stool, driven by a desire to avoid uncomfortable defecation experiences stemming from fear. In younger children, the onset of constipation might coincide with the initiation of potty training. During this time, pressure exerted by caregivers to maintain control over bowel movements or the use of inappropriate techniques, like employing baths lacking sufficient leg support, can trigger the retention of stool. This conduct results in withholding of feces, which prompts the colon to absorb excess water, leading to the formation of firm stools. Conversely, within the initial years of life, a sudden episode of constipation due to dietary changes can lead to the passage of dry and firm stools, subsequently causing painful defecation. The Diagnostic criteria for Functional Constipation were redefined in Rome IV (5).

#### 4.2.1 Gut-brain-microbiota axis and Functional Constipation

Vriesman et al. in their review underscore the comprehensive management of functional constipation in children and adults. They describe functional constipation as a multifactorial condition with genetic, lifestyle, and psychological contributions. The review emphasizes the connection between psychological factors and functional constipation, highlighting the interaction through the efferent and afferent pathways of the gut-brain axis. This approach aligns with the broader understanding that functional constipation is not solely based on intestinal dysfunctions but is also influenced by psychological and neuro-gastro-enterological factors. Together, these perspectives provide a comprehensive view that integrates clinical management and the latest research on gut microbiota in functional constipation (23).

Expanding on this perspective, the study conducted by Wang et al. delves deeper into the critical role of the gut microbiome and its metabolites in the pathogenesis of functional constipation. Recent evidence from both human and animal studies highlights a robust association between gut microbiota and functional constipation, elucidated through the brain-gut-microbiome axis. This intricate interplay involves the modulation of gut functions by gut microbiota through the production of metabolites from bacterial fermentation. Among these metabolites, short-chain fatty acids (SCFAs), secondary bile salts (BAs), and methane emerge as pivotal players. Notably, they have the capacity to initiate the release of gut hormones from enteroendocrine cells (EECs), including 5-hydroxytryptamine (5-HT), peptide YY (PYY), and glucagon-like peptide-1 (GLP-1). Subsequently, these gut hormones exert influence over gut sensation, secretion, and motility. Their primary mode of action involves the activation of specific receptors distributed on smooth muscle cells, enteric neurons, and epithelial cells. This detailed cascade underscores the multifaceted role of gut microbiota and their metabolites in shaping gut physiology and contributing to the development of functional constipation (24).

#### 4.2.2 Microbiome composition and Functional Constipation in children

Changes in the composition of the intestinal microbiota are regarded as a contributing factor in pediatric Functional Constipation. In a study by Meij et al., microbial composition and diversity in children with Functional Constipation (Rome III criteria) were examined in comparison to healthy controls. They discovered that the most prominent species included *Bacteroides fragilis*, *Bacteroides ovatus*, *Bifidobacterium longum*, and *Parabacteroides* species, all of which exhibited a correlation with a higher incidence of Functional Constipation. In contrast, was linked to a reduced occurrence of Functional Constipation (25).

In another study conducted by Mancabelli et al., a microbial profile analysis based on 16S rRNA was performed on stool samples collected from individuals diagnosed with Functional Constipation, and the results were compared to those of healthy subjects. The findings indicated that the gut microbiota of individuals with Functional Constipation lacked members associated with Bacteroides, Roseburia, and Coprococcus. Additionally, it was observed that the microbiomes of individuals with Functional Constipation exhibited a significant abundance of genes involved in hydrogen production, methanogenesis, and glycerol degradation. The study concludes that the identified discrepancies in bacterial composition and metabolic capacities could have a notable impact on the emergence of symptoms associated with Functional Constipation (26).

Zhu et al. employed 16S rRNA gene sequencing to demonstrate that children diagnosed with Functional Constipation exhibited a notably reduced presence of *Bacteroidetes*, particularly Prevotella, alongside an increased abundance of several *Firmicutes* species, inclusive of *Lactobacillus*. Their findings also indicated that the levels of *Bifidobacterium* species remained unchanged.

Khalif et al. conducted an examination of the gut microbiota in adults experiencing functional constipation, revealing a decline in Bifidobacterium, Lactobacillus, Bacteroides, and Clostridium species, accompanied by an elevation in Enterobacteriaceae, such as *Escherichia coli*, along with *Staphylococcus aureus* and fungal populations (27). On the other hand, another study indicated that adults diagnosed with Functional Constipation displayed notably reduced levels of *Bifidobacterium* and *Bacteroides* (28). While our focus is primarily on children, it's crucial to acknowledge insights from studies in adults. Notably, the existing evidence in pediatric populations is limited, and interventions explored in adults might pave the way for future research in children. For instance, the observed microbiota changes in adults could serve as a valuable reference point for understanding potential variations in the pediatric population.

In summary, Functional Constipation emerges as a puzzle with multiple contributing pieces, wherein the disruption of the microbiota emerges as a plausible piece of this puzzle. Yet, the role of the gut's microbial inhabitants remains enshrouded in uncertainty; findings among children and adults diverge, keeping the mystery alive. The stage demands more investigations to unravel the microbial threads woven into the intricate tapestry of childhood constipation's underlying mechanisms.

#### 4.2.3 Studies of probiotics in Functional Constipation

In the context of constipation, evidence in adults suggests that certain probiotic strains might effectively alleviate constipation; however, results in children remain inconclusive. Concrete recommendations have been established for the adult population. However, in pediatrics, position papers, such as the one by the ESPGHAN Special Interest Group on Gut Microbiota and Modifications regarding Probiotics for the Management of Pediatric Gastrointestinal Disorders, do not endorse the use of probiotics as a sole or adjunct treatment for Functional Constipation in children due to the lack of demonstrated efficacy in clinical studies (16). Despite studies involving probiotic strains for children's constipation, further research is essential to potentially integrate these recommendations into guidelines such as those of ESPGHAN or WGO.

An illustrative example is found in the observational pilot study conducted by Astó et al. (29), which explored the effects of *Bifidobacterium longum* KABP042 and *Pediococcus pentosaceus* KABP041 strains in infants diagnosed with FGIDs, including infant colic and functional constipation. This study revealed that the probiotic formula was well-tolerated and led to a significant reduction in FGID severity after only a 14-day treatment with the two strains. Nonetheless, to solidify these findings and uncover deeper insights, additional comprehensive investigations are imperative.

Table 2 includes a list of studies conducted with probiotics for the management of Functional Constipation in children.

**Table 2:** Studies of probiotics in constipation in children.

Autor	Intervention	No patients (age)	Resulted
Banaszkiewicz and Szajewska (42)	*LGG* 2 × 10^9^ CFU *+ Lactulose*	84 (2–16 years)	Non-significant difference
Bu et al. (43)	*L. casei rhamnosus* Lcr35 8 × 10^8^ CFU	27 (<10 years)	Higher defecatory frequency, higher percentage of success in treatment, lower use of glycerin enema
Coccorullo et al. (44)	*L. reuteri* DSM17938 1 × 10^8^	44 (>6 months)	Positive effect on bowel frequency
Guerra et al. (45)	Goat yogurt (1 ml) containing *B. longum* 10^9^ CFU, plus yogurt starters	59 (5–15 years)	Improves stool consistency
Sadeghzadeh et al. (46)	*L. casei* PXN37, L. rhamnosus PXN54, *S. thermophilus* PXN66, *B. breve* PXN25, *L. acidophilus* PXN35, *B. infantis* PXN27, *L. bulgaricus* PXN39, 1 × 10^9^ CFU + Lactulose	48 (4–12 years)	At the end of the first week fecal incontinence and abdominal pain improved significantly in the intervention group; At the end of the fourth week, this difference was not significant.
Wojtyniak et al. (47)	*L. casei rhamnosus Lcr35, 8 × 10^8^ CFU*	94 (<5 years)	Non-significant difference
Wegner et al. (48)	L. reuteri DSM17938 *10^8^*CFU + Macrogol	129 (3–17 years)	Non-significant difference
Kubota et al. (49)	Group A (*n* = 20) *L. reuteri* DSM17938 and lactose hydrate as a placebo of MgO; group B (*n* = 19) *L. reuteri* DSM17938 and MgO; and group C (*n* = 21) a placebo of *L. reuteri* DSM17938 and MgO.	60 (6 months-6 years)	*L. reuteri* DSM17938 and MgO were both effective in the management of functional constipation in young children. MgO caused an imbalance in the gastrointestinal microbiome, which was not the case in the probiotic group.
Žaja et al. (50)	Patients with anorexia nervosa and constipation *L. reuteri* DSM17938 *10^8^* CFU	31 (10 -18 years)	Increasing stool frequency and nutritional recovery in childhood AN after six months

CFU, colony forming units; LGG, *Laticaseibacillus rhamnosus*; GG, MgO magnesium oxide.

### 4.3 Functional abdominal pain disorders in childhood

The Functional Abdominal Pain Disorders in children and adolescents were classified under the H2 category of Rome IV, and their diagnostic criteria were defined. This category encompasses conditions like Functional Dyspepsia, Irritable Bowel Syndrome (IBS), Abdominal Migraine, and Functional Abdominal Pain Not Otherwise Specified (6). A notable diagnostic hurdle arises from the possible overlap among these four gut-brain interaction disorders.

#### 4.3.1 Functional Dyspepsia

##### 4.3.1.1 Gut-Brain Interaction Axis in Functional Dyspepsia

As many as half of individuals with unexplained dyspepsia in the general population attribute stress as a catalyst for their symptoms. It is suggested that the impact of stress could be channeled through heightened permeability and immune activation. Emerging preclinical investigations suggest that stress might trigger gut dysbiosis, influencing central nervous system activities and behaviors. These observations have given rise to the notion of a two-way Gut-Brain-Axis, although substantiating evidence remains limited (30).

##### 4.3.1.2 Microbiota in Functional Dyspepsia

Despite advances in multiomic technology, there are few studies in pediatrics on the microbiota in Functional Dyspepsia. The fecal microbiota does not always reflect what happens with the intestinal microbiota, especially with what is related to the duodenal microbiota where there is a lower density and greater diversity of bacteria with a predominance of Gram-positive aerobes.

Zhou and colleagues conducted a review exploring the role of the microbiota in Functional Dyspepsia. Within their findings, three human studies with limited patient samples indicated a decrease in *Actinomycete*, *Atopobium collin*, *Leptotrichia trevisan*, *Prevotella*, and *Veillonella abundance*. Conversely, an increase was observed in the phylum *Firmicutes* and *Streptococcus*, along with a higher relative abundance of *Bifidobacterium* and *Clostridium*. This study underscores the clear presence of evidence depicting alterations in the relative abundance and composition of the gastrointestinal tract's microbiota, which significantly contributes to the development of Functional Dyspepsia (31).

#### 4.3.2 Irritable Bowel Syndrome (IBS)

##### 4.3.2.1 Gut-Brain Interaction Axis in IBS

IBS is recognized as a disorder of the brain-gut axis, where individual symptoms such as diarrhea, constipation, pain severity, and psychosocial distress reflect the affected components and their extent. Children with IBS may exhibit varying forms of visceral hypersensitivity, potentially linked to psychological distress like anxiety and depression. Elevated mucosal proinflammatory cytokines could stem from postinfectious IBS. While alterations in gut microbiome have been shown, their causal relationship to IBS remains unclear. Children with IBS often report increased stress, anxiety, depression, and emotional issues, and early-life noxious events like surgery can heighten the risk of developing IBS in childhood (6).

##### 4.3.2.2 IBS and microbiota

Amidst the numerous factors contributing to IBS, dysbiosis and its interplay with the brain-gut axis hold significant relevance. The composition of gut microbiota in healthy children and pediatric IBS sufferers remains relatively undefined; studies conducted in adults have suggested the potential involvement of gastrointestinal microbiota in IBS.

Saulnier et al. conducted a study employing 16S rRNA gene sequencing to analyze samples from children diagnosed with IBS based on Rome III pediatric criteria, as well as samples from healthy children with an age range between 7 and 12 years. Microbiomes linked to pediatric IBS displayed a notable elevation in the proportion of class γ Proteobacteria, with *Haemophilus parainfluenzae* being a significant member of this group. Additionally, a novel *Ruminococcus*-like microorganism was identified in association with IBS, suggesting the potential value of microbial discovery in understanding gastrointestinal disorders. Moreover, a higher frequency of pain was found to correlate with an increased presence of various bacterial taxa belonging to the genus *Alistipes* (32).

Rigsbee et al. investigated the quantitative composition of the intestinal microbiota in children primarily suffering from diarrhea-predominant IBS. Fecal samples were collected from healthy pre- and adolescent volunteers, age range 11–18 years, and children newly diagnosed with IBS-D, age range 8–18 years. Their study revealed notable differences in the abundance of several genera between healthy individuals and those diagnosed with IBS. Among these distinctions, the levels of Veillonella, Prevotella, Lactobacillus, and Parasporo increased in children with IBS, while members of Bifidobacterium and Verrucomicrobium were found to be less abundant in this group (33).

In a comprehensive literature analysis conducted by Pittayanon et al., the majority of the studies focused on adult individuals with various subtypes of IBS. It was observed that in comparison to control groups, patients with IBS exhibited an increase in the abundance of certain microbial groups, such as the Enterobacteriaceae family (belonging to the Proteobacteria phylum) and the Lactobacillaceae family, as well as the Bacteroides genus. Conversely, a decrease was found in the levels of uncultured *Clostridiales* I, the Faecalibacterium genus (including *Faecalibacterium prausnitzii*), and the Bifidobacterium genus in patients with IBS (34).

Another study in adults, which also examined the microbiome of both IBS patients and healthy controls, discovered that IBS patients exhibited significantly lower microbial diversity, which was correlated with a reduced relative abundance of bacteria that produce butyrate. The presence of the methanogen *Methanobrevibacter smithii* has been linked to the constipation subtype of IBS, as methane production is associated with slow intestinal transit. On the other hand, the diarrhea subtype of IBS has been associated with a decrease in butyrate-producing bacteria like Faecalibacterium and Ruminococcus, as well as a reduction in *Clostridium leptum*. This particular bacterium is capable of converting primary bile acids into secondary ones, which has implications for fecal consistency (35).

#### 4.3.3 Functional Abdominal Pain Not Otherwise Specified (FAP-NOS)

The term “Functional Abdominal Pain-Not Otherwise Specified FAP-NOS” in Rome IV replaces the terms “Functional Abdominal Pain” and “FAPS” from Rome III. Studies differentiating FAP-NOS from IBS suggest that rectal hypersensitivity is generally absent in children with FAP-NOS, unlike in those with IBS. Children with FAP-NOS display lower antral contractions and slower emptying rates of liquid meals compared to healthy controls, though the significance remains uncertain. Psychological distress is linked to chronic abdominal pain in youth, with associations to stressful life events like parental divorce, hospitalization, bullying, and childhood abuse. Outcomes of FAPDs are influenced by how a child and their family manage pain.

##### 4.3.3.1 Functional Abdominal Pain Not Otherwise Specified and microbiota

The microbiome analysis conducted by Abomoelak et al. in pediatric patients with Functional Abdominal Pain (FAP) according to the Rome III criteria revealed that the phylum *Bacteroidetes* exhibited higher levels compared to the healthy control group, while the phylum *Firmicutes* was found to be lower in individuals with FAP. Additionally, the phylum *Verrucomicrobia* displayed a higher abundance in the control group than in those with FAP. Notably, the diversity of the microbiome was also observed to be higher in the control group than in individuals with FAP. This study highlights the altered state of the gut microbiome in individuals with FAP compared to the control group (36).

#### 4.3.4 Probiotic studies in Functional Abdominal Pain

Similar to the treatment of Infant Colic, numerous controlled clinical studies have examined the impact of probiotics on Functional Abdominal Pain and IBS. Among the probiotic strains, *Lactocaseibacillus rhamnosus* GG ATCC 53103 (LGG®) and *Limosilactobacillus reuteri* DSM 17938 have emerged as the most extensively studied (37). These strains have also secured their place on the roster of probiotics with evidence for Functional Abdominal Pain and IBS, as highlighted in the 2023 World Gastroenterology Organisation's Probiotics and Prebiotics Guidelines (19).

As per ESPGHAN recommendations, healthcare professionals may consider suggesting *Lacticaseibacillus rhamnosus* GG ATCC 53103 (LGG®) at a dose ranging from 10^9^ CFU to 3 × 10^9^ CFU twice daily. This supplementation is proposed to potentially reduce the frequency and intensity of pain in children with IBS. The certainty of evidence is deemed moderate, and the degree of recommendation stands as weak. Similarly, healthcare professionals may advise the use of *Limosilactobacillus reuteri* DSM 17938 at a dose ranging from 10^9^ CFU to 10^9^ CFU twice daily. *Limosilactobacillus reuteri* DSM 17938 (at a dose of 10^8^ CFU at 2 × 10^8^ CFU/day) can be used for the reduction of pain intensity in children with Functional Abdominal Pain Not Otherwise Specified, with the certainty of evidence categorized as moderate and the recommendation level being low (18).

Other individual studies have showcased promising outcomes. For instance, Vázquez-Frias et al. conducted a trial involving pediatric patients with IBS (Rome IV criteria) lasting at least 2 months. In this study, participants were given either B. clausii OC NR SIN T or a placebo, administered once daily for the duration of 2 months. The study did not reveal significant efficacy differences between *B. clausii* and placebo when combined with conventional treatment. Nonetheless, the observed treatment response was 80%, prompting consideration for a larger-scale study to solidify these findings (38).

In 2010, Guandalini et al. carried out a multicenter, randomized, placebo-controlled, double-blind, crossover study, which demonstrated that VSL#3, a probiotic blend, is not only safe but also more effective than a placebo in alleviating symptoms and enhancing the quality of life in children with IBS (39).

In a distinct investigation, children who met the criteria for IBS according to Rome III criteria were involved in a trial conducted in Mumbai. These children were provided with either *Bacillus coagulans* Unique IS2 chewable tablets or a placebo once a day for a duration of eight weeks, followed by a two-week follow-up period. The treated group exhibited a more pronounced reduction in pain scores, alongside significant enhancements in stool consistency, reduced abdominal discomfort, bloating, staining, urgency, incomplete evacuation, and passage of gas. Significant enhancements were also noted in the level of contentment with bowel habits and the overall evaluation of relief among the group treated with B. coagulans Unique IS2 (40).

## 5 Conclusions

While the exploration of the human microbiome and its genuine influence on health is still in its early stages, an increasing body of evidence has indicated that the microbiota has a substantial role in the pathogenesis of Disorders of Gut-Brain Interaction (DGBIs). The profile that characterizes dysbiosis in DGBIs is being revealed and may be useful as a biomarker for early diagnosis and treatment.

The application of probiotics emerges as a promising strategy in pediatric care. While our focus in this review has primarily centered on probiotics, it is imperative to acknowledge the potential microbiota-modifying bioactives like prebiotics, synbiotics, postbiotics, and LBP to the management of pediatric disorders of gut-brain interaction. These bioactives, although not mentioned, represent diverse avenues for further exploration and research, and their specific roles warrant deeper investigation. Future studies should delve into the mechanisms of action, efficacy, and potential synergies among these microbiota-modifying interventions to enhance our understanding and broaden the therapeutic landscape for pediatric patients.

Table 1 summarizes the evidence-based recommendations available to 2022 of the use of probiotics in DGBIs from the ESPGHAN Special Interest Group on Gut Microbiota and Probiotic Modifications for the Management of Pediatric Gastrointestinal Disorders and from World Gastroenterology Organisation Global Guidelines forProbiotics and Prebiotics.

This diagram illustrates the Microbiota-Gut-Brain Axis, demonstrating the bidirectional connections among the gut, microbiota, and brain. An altered microbiota, associated with a compromised gut barrier and activated mucosal immune response, initiates the liberation of molecules with inflammatory and neuroactive properties into the bloodstream. These substances subsequently reach the brain, triggering cognitive and behavioral alterations. Conversely, external factors like stress can disrupt mucosal immunity, the gut microbiota, and barrier function, potentially leading to gut dysfunction. This visual emphasizes the intricate interplay linking the gut, microbiota, and brain, highlighting both the pathways of communication from gut to brain and from brain to gut (41).

## Grupo de investigación del caribe y centroamérica para la Microbiota, probióticos y prebióticos GICCAMPP

Ana Teresa Abreu, Active Member, Board Member. Rosa Acevedo, Founding Member, Vice-President. Marie Claire Arrieta, Honorary Member. Roderick Bejarano, Founding Member. Gisselle Fernández, Active Member, Treasurer. Rodrigo Vázquez Frias, Founding Member, Board Member. Filipa Godoy, Advisory Member. Marco Luis Herrera, Active Member, Board Member. Jorge Martínez, Active Member, Secretary. *León de Mezerville, Founding Member, Honorary Member*. Jorge Palacios, Founding Member, Board Member. Randall Quesada, Founding Member, Board Member. Dimas Rosa, Founding Member, Past-President. Carolina Ortiz, Founding Member, President. Catalina Ortiz, Advisory Member. Roberto Arturo Zablah, Founding Member.
